# Cross sectional study in China: fetal gender has adverse perinatal outcomes in mainland China

**DOI:** 10.1186/s12884-014-0372-4

**Published:** 2014-10-26

**Authors:** Lei Hou, Xin Wang, Guanghui Li, Liying Zou, Yi Chen, Weiyuan Zhang

**Affiliations:** Department of Obstetrics, Beijing Obstetrics and Gynecology Hospital, Capital Medical University, Beijing, 100026 China

## Abstract

**Background:**

The association between fetal gender and pregnancy outcomes has been thoroughly demonstrated in western populations. However, this association has not been thoroughly documented in China. The primary objective of the present study is to determine whether the association of adverse pregnancy and labour outcomes with male fetuses applies to the Chinese population.

**Methods:**

This cross-sectional hospital-based retrospective survey collected data from thirty-nine hospitals in 2011 in mainland China. A total of 109,722 women with singleton pregnancy who delivered after 28 weeks of gestation were included.

**Results:**

Of these pregnancies, the male-to-female sex ratio was 1.2. The rates of preterm birth (7.3% for males, 6.5% for females) and fetal macrosomia (8.3% for males, 5.1% for females) were higher for male newborns, whereas fetal growth restriction (8.0% for females, 5.4% for males) and malpresentation (4.3% for females, 3.6% for males) were more frequent among female-bearing mothers. A male fetus was associated with an increased incidence of operative vaginal delivery (1.3% for males, 1.1% for females), caesarean delivery (55.0% for males, 52.9% for females), and cephalopelvic disproportion/failure to progress (10.0% for males, 9.2% for female). Male gender was also significantly associated with lower Apgar scores (<7 at 5 min, adjusted odds ratio 1.3, 95% CI 1.0-1.6), as well as a neonatal intensive care unit admission and neonatal death, even after adjustments for confounders (adjusted odds ratio 1.3, 95% CI 1.1-1.5, adjusted odds ratio 1.4, 95% CI 1.1-1.8).

**Conclusion:**

We confirm the existence of obvious neonatal gender bias and adverse outcomes for male fetuses during pregnancy and labour in our population. Further research is required to understand the mechanisms and clinical implications of this phenomenon.

**Electronic supplementary material:**

The online version of this article (doi:10.1186/s12884-014-0372-4) contains supplementary material, which is available to authorized users.

## Background

The increased risk for operative delivery among mothers carrying male fetuses was first documented by Hall and Carr-Hill more than a quarter of a century ago [1]. The predominance of a broad range of adverse neonatal outcomes in males has been noted, most notably preterm labour, premature rupture of membranes (PROM), hypertension disorder complicating pregnancy (HDCP), gestational diabetes mellitus (GDM) and fetal macrosomia [2,3]. Women carrying male fetus also have a higher incidence of cord complications, fetal distress, labour dystocia, operative delivery and caesarean delivery [2-5].

The factors associated with adverse pregnancy and neonatal outcomes are complex and depend on race, ethnicity, environmental conditions and biology. It is well documented that race and maternal environment affect fetal growth; for example, infants born to African-American mothers are smaller, and they have higher rates of growth restriction and preterm delivery compared with infants born to white mothers [6]. The majority of studies focusing on the relationship between fetal sex and pregnancy outcomes were performed in western countries; there are limited reports published in Asia. Moreover, there are conflicting results between different countries. Studies have drawn similar conclusions that women carrying a male fetus have a high risk of arrested labour and elective caesarean [5,7]. A study in Israel did not find an increased risk of caesarean section in women carrying male fetuses [8]. Differences between ethnicity and obstetrics care in various populations may account for this discrepancy between studies. However, the relatively low frequency of severe outcomes complicates the appropriate assessment of whether fetal sex is associated with increased risk of neonatal outcomes; thus, these studies require a large sample size. We sought to conduct more detailed studies in mainland China to better understand the basic mechanisms of how fetal sex influences pregnancy and neonatal outcomes.

## Methods

**Figure 1:**
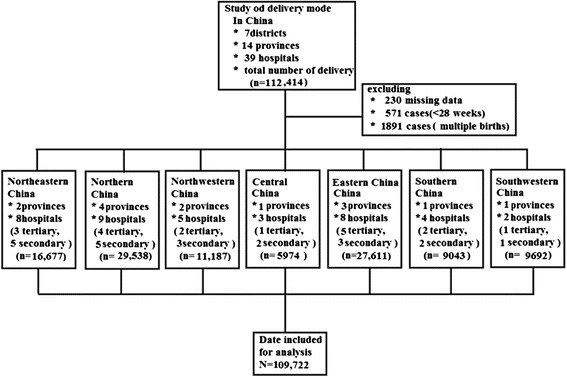
**A retrospective analysis that was performed on 112414 babies, covering 39 hospitals of different levels in mainland China.**

The current report was performed in mainland China. It is a multi-centred, large-sample, cross-sectional study. All of the participating facilities are members of the obstetrics cooperative centre, which consists of a total of 39 public hospitals of different levels and covers 14 provinces, municipalities, and autonomous regions within China (Beijing, Shanghai, Jilin, Liaoning, Jiangsu, Sichuan, Shanxi, Hubei, Guangdong, Hebei, Inner Mongolia, Shandong, Shanxi, and Xinjiang Figure 1). Twenty of the hospitals were tertiary care hospitals, and nineteen were secondary care hospitals. Private hospitals were not included because private hospitals only accounted for 11.1% of all inpatients in China in 2012 [9].

Chinese hospitals are divided by the government into primary, secondary, and tertiary hospitals according to the Hospital Classification Guidelines based on the tasks and functions of the hospital, the facility’s technology, the quality of medical services, and the overall level of scientific management. Tertiary hospitals provide the highest level of medical service. Primary care facilities include hospitals and community-based health care facilities that provide health care, disease prevention, rehabilitation, and other basic services to communities. Most primary care hospitals only provide low-level care, are not equipped to perform caesarean sections, and lack an emergency obstetric or neonatal care unit. Statistical Yearbook 2013 showed that the number of inpatients in tertiary and secondary hospitals accounted for 94.4% of all inpatients in China in 2012 [9]; therefore, primary care hospitals were not included in the present study. All facilities were selected from tertiary and secondary hospitals.

Women giving birth at the selected facilities between January 1st and December 31st, 2011 were included. The inclusion criteria were women with singleton pregnancies who had completed 27 weeks of gestation (The perinatal period was defined as the period after the birth of an infant born at 28 weeks and ending at 28 days after birth in China). The following clinical characteristics and obstetrical risk factors were analysed: maternal age, gravidity, parity, obesity, gestational age, GDM, DM (diabetes mellitus), HDCP, PROM, placenta previa, placenta abruption, oligohydramnios, fetal growth restriction (FGR), breech, shoulder presentation and NRFHT (non-reassuring fetal heart rate tracing). The outcome of labour and the main indications for caesarean delivery were assessed. Inpatient neonatal outcomes were investigated, including the birth weight, Apgar score, admission to a neonatal intensive care unit (NICU) and neonatal death. Neonatal death was defined as death during the first 28 days of life in the hospital. There was no follow-up after they discharged from the hospital.

Operative vaginal delivery included forceps delivery, vacuum extraction delivery and breech extraction. Medical indications for caesarean delivery were defined by Williams Obstetrics (23rd edition). A maternal request for caesarean was defined as a primary caesarean delivery upon maternal request in the absence of any maternal or fetal indications.

Preterm birth was defined as delivery after at least 28 weeks of gestation but no more than 37 weeks of gestation. Fetal macrosomia was defined as an estimated birth weight above 4 kilograms. Malpresentation included breech presentation, face presentation, transverse lie, and unstable lie. FGR refers to a fetus with a birth weight less than the 10th percentile. An amniotic fluid index (AFI) <5 cm was used as a cut-off to define oligohydramnios. GDM refers to various degrees of abnormal glucose tolerance that occur in pregnancy. The following blood glucose values were used to diagnosis GDM: the fasting level and the levels 1 and 2 h after an Oral Glucose Tolerance Test (OGTT) with cut-offs of 5.1, 10.0, and 8.5 mmol/L (92, 180, and 153 mg/dl), respectively. If any measurement reached or exceeded these threshold values, the patient was diagnosed with GDM. Obesity was defined as a body mass index (BMI) ≥28 (based on recommendations by the Group of China Obesity Task Force of the Chinese Ministry of Health). Prior uterine surgery was defined as a history of caesarean delivery (CD) or previous uterine surgery, such as myomectomy.

The procedures of this study received ethics approval from every Human Ethics Committees of participating hospital (Additional file 1). We obtained all the individual-level data from medical records and did not identify the participants. To ensure patient privacy, the data did not include the mother’s name, phone number, home address, or other personal information. Institutional informed consent was obtained from the responsible authority of each of the participating health facilities. Investigators from each province, municipality, and autonomous region were responsible for training personnel in data entry. The investigators reviewed the medical records, and the trained staff collected and entered the data for computer-based statistical analysis.

Statistical analyses were performed using the SPSS statistics software version 18.0. Data are presented as percentages or median with inter-quartile range. Pearson’s chi-squared test of independence was used to identify whether maternal disease, term status, modes of delivery and neonatal outcomes were correlated with fetal sex. The Mann–Whitney U test was used for the maternal age, gestational age and birth weight. P values were considered to be significant at values < 0.05. Univariate analysis was performed separately for the assessment of the association between the characteristics of the facilities, mothers and babies individually with each neonatal outcome. The variables that were significantly associated with the outcome in the univariate analysis (p < 0.05) were successively included in a multivariate model. In the multivariate analysis, the risks of neonatal outcomes associated with fetal sex are presented using adjusted ORs with the corresponding 95% CIs. For institutional characteristics, the risk of neonatal adverse outcomes was higher in the secondary hospitals, suggesting an ecological relationship between the strength of the health system, urbanisation, and development status; thus, we treated the hospital grade as a confounding factor to be adjusted.

## Results

A total of 112,414 deliveries occurred during the study period. Records with data missing for the delivery mode (230 cases), a gestation period of less than 28 weeks (571 cases), and multiple fetuses (1891 cases) were excluded from the study, leaving 109,722 complete records for the study.

### Pregnancy outcomes

From the selected 109,722 deliveries during the one-year period, 60,028 (54.7%) had male neonates, and 49,694 (45.3%) had females, with a male-to-female sex ratio of 1.2 (Table 1). The mean maternal age was similar between the two groups. The women aged 25–34 years were more likely to have a female fetus (67.8% vs 67.0%, OR: 1.0, 95% CI: 0.9-1.0, p < 0.01). A large percentage of newborn males compared with females were born to the women whose ages were up to thirty-five years (10.3% vs 9.6%, OR = 1.1, 95% CI: 1.0-1.1, p < 0.001). Primiparous women are more common in China because of the one-child family policy. In our study, 81.4% of all deliveries were primiparous women. The percentage of females born to primiparous mothers was greater than the percentage of males (82.8% vs 80.3%, p < 0.001) (Table 1). Women with high gravidity and parity were more likely to deliver male fetuses; both of these differences were significant (p < 0.001). There were no significant differences between the groups regarding residence location or obesity.

**Table 1:** **Characteristics of study populations**

	**Male infants (n = 60028)**	**Female infants (n = 49694)**	**Ratio**	**P**
	**n (%)**	**n (%)**		
Maternal age (years)	28 (25–31)	28 (25–31)		0.48
≤24	13894 (23.3%)	11371 (23.0%)	1.22	0.24
25-34	39532 (66.4%)	33249 (67.4%)	1.19	<0.001
≥35	6105 (10.3%)	4736 (9.6%)	1.29	<0.001
Gravidity	2 (1–2)	1 (1–2)	-	<0.001
Parity	1 (1–1)	1 (1–1)	-	<0.001
Obesity	8361 (17.1%)	7109 (17.2%)	1.18	0.07
Primiparous	48176 (80.3%)	41130 (82.8%)	1.17	<0.001

835 missing cases for maternal ages; 19578 missing cases for maternal weight and height.

Quantitative variables are expressed as median (25th percentile–75th percentile).

The overall incidence of GDM, DM, HDCP, preeclampsia, PROM, placenta previa, placenta abruption, oligohydramnios and NRFHT showed no significant relationship with fetal sex. Malpresentation was more frequent among the female-bearing mothers (p < 0.001). FGR was also more frequent among female-bearing mothers (8.0% vs 5.4%,), even after adjusting for significant confounders (adjusted for resident, maternal age, education, HDCP, GDM, DM, placenta previa, placenta abruption, other medical disorders and malpresentation) (OR = 1.5, 95% CI: 1.4-1.6, p < 0.001) (Table 2).

**Table 2:** **Comparison of pregnancy-related risk factors**

	**Male infants (n = 60028)**	**Female infants (n = 49694)**	**Ratio**	**P**
	**n (%)**	**n (%)**		
GDM	2778 (4.6%)	2254 (4.5%)	1.23	0.47
DM	67 (1.1‰)	65 (1.3‰)	1.03	0.36
HDCP	2593 (4.3%)	2217 (4.5%)	1.17	0.25
Preeclampsia, eclampsia	1777 (3.0%)	1549 (3.1%)	1.15	0.13
PROM	9096 (15.2%)	7509 (15.1%)	1.21	0.85
Placenta previa	742 (1.2%)	586 (1.2%)	1.27	0.39
Placenta abruption	311 (0.5%)	264 (0.5%)	1.18	0.76
Oligohydramnios	2286 (3.8%)	1891 (3.8%)	1.21	0.98
FGR	3190 (5.4%)	3918 (8.0%)	0.81	<0.001
Malpresentation	2134 (3.6%)	2145 (4.3%)	0.99	<0.001
NRFHT	4944 (8.2%)	4139 (8.3%)	1.19	0.58

GDM, gestational diabetes mellitus; HDCP, hypertension disorder complicating pregnancy; DM, preexisting diabetes mellitus; PROM, premature rupture of membranes; Malpresentation, including breech presentation, face presentation, transverse lie, and unstable lie; FGR, Fetal Growth Restriction; NRFHT, non-reassuring fetal heart rate Tracing. 1744 missing cases for neonatal weight.

### Labour outcomes

For vaginal deliveries, male fetuses had a higher rate of operative intervention (1.3% vs 1.1%, OR = 1.2, 95% CI: 1.1-1.3, p < 0.001). Male fetuses also had a higher chance of caesarean delivery than females (55.0% vs 52.9%, OR = 1.1, 95% CI: 1.1-1.1, p < 0.001). In general, the top three indications for caesarean delivery were maternal request, cephalopelvic disproportion/failure to progress and fetal distress, which accounted for 61.8% of the caesarean deliveries. Of the 33,029 male-bearing women who underwent caesarean delivery, the rate of maternal request was higher than that for female-bearing women (17.1% vs 16.6%, p = 0.05). The rate of cephalopelvic disproportion/failure to progress was also higher in the male-bearing women than in the female-bearing women (10.0% vs 9.2%, OR = 1.1, 95% CI: 1.1-1.2, p < 0.001); these associations persisted after adjustments for the birth weight, which was higher in males than in females (OR = 1.1, 95% CI: 1.0-1.1, p < 0.001). Consistent with the higher prevalence of malpresentation for female-bearing women, the rate of caesarean section for malpresentation was higher significantly in the female-bearing women than in the male-bearing women. Consistent with the higher prevalence of macrosomia in the male-bearing women, the rate of caesarean section for suspected macrosomia was higher significantly in the male-bearing women than in the female-bearing women. The rate of caesarean delivery for fetal distress showed no significant difference between male and female fetuses (Table 3).

**Table 3:** **Comparison of delivery mode and indication for cesarean delivery**

	**Males (n = 60028)**	**Females (n = 49694)**	**Ratio**	**P**
	**n (%)**	**n (%)**		
Spontaneous delivery	26239 (43.7%)	22878 (46.0%)	1.15	<0.001
Operative vaginal delivery^a^	760 (1.3%)	534 (1.1%)	1.42	<0.01
Cesarean delivery	33029 (55.0%)	26282 (52.9%)	1.26	<0.001
Maternal request	10243 (17.1%)	8263 (16.6%)	1.24	0.05
Cephalopelvic disproportion/Failure to progress	6032 (10.0%)	4591 (9.2%)	1.31	<0.001
Fetal distress	4130 (6.9%)	3400 (6.8%)	1.21	0.80
Previous uterine surgery	3545 (5.9%)	2689 (5.4%)	1.32	<0.001
Malpresentation	1710 (2.8%)	1742 (3.5%)	0.98	<0.001
Suspected macrosomia	2227 (3.7%)	1188 (2.4%)	1.87	<0.001
Oligohydrammios	1203 (2.0%)	981 (2.0%)	1.23	0.72
Preeclampsia/eclampsia	1105 (1.8%)	1011 (2.0%)	1.09	<0.05
Later pregnancy bleeding	823 (1.4%)	727 (1.5%)	1.13	0.20

^a^Operative vaginal delivery includes forceps delivery, vacuum extraction delivery and breech extraction.

There were no differences between the two groups in mean gestational age, but the incidence of preterm birth was significantly higher in the male-bearing women compared with female-bearing women, with a male-to-female ratio of 1.36 (OR = 1.1, 95% CI: 1.1-1.2, p < 0.001) even after adjustment for significant confounders (adjusted for hospital grade, resident, maternal age, maternal education, gravity, parity, bad obstetrics history, HDCP, GDM, DM, PROM, placenta previa, placenta abruption, other medical disorders, as well as malpresentation. OR = 1.2, 95% CI: 1.1-1.2, p < 0.001). The male birth weights were significantly higher than the female birth weights (p < 0.001), and male newborns were more likely to have macrosomia (OR = 1.7, 95% CI: 1.6-1.7, p < 0.001) (Table 4).

**Table 4:** **Comparison of neonatal outcomes**

	**Male infants (n = 60028)**	**Female infants (n = 49694)**	**Ratio**	**P**
	**n (%)**	**n (%)**		
**Gestational age (GA)**				
Mean GA (weeks)	39 (38–40)	39 (38–40)	-	<0.001
Preterm	4381 (7.3%)	3233 (6.5%)	1.36	<0.001
Term	51576 (85.9%)	43063 (86.7%)	1.20	<0.001
Postterm	4071 (6.8%)	3398 (6.8%)	1.20	0.71
**Neonatal outcome**				
Birth weight (BW)				
Mean BW (g)	3350 (3040–3640)	3250 (2980–3520)	-	<0.001
<2500 g	3548 (6.0%)	3333 (6.8%)	1.06	<0.001
2500-4000	51628 (87.9%)	43852 (89.0%)	1.18	<0.001
>4000 g	4852 (8.3%)	2509 (5.1%)	1.93	<0.001
Apgar < 7 at 5 min	294 (0.5%)	193 (0.4%)	1.5	<0.05
NICU	624 (1.0%)	455 (0.9%)	1.37	<0.05
Neonatal death	241 (0.40%)	124 (0.25%)	1.94	<0.01

NICU, neonatal intensive care unit Quantitative variables are expressed as median (25th percentile–75th percentile).

1744 missing cases for neonatal weight. 5346 missing cases for Apgar scores at 5 minutes, 35 missing cases for neonatal death.

In our study, male fetuses had higher rates of low Apgar scores at 5 min (0.5% vs 0.4%, OR = 1.3, 95% CI: 1.1-1.5, p < 0.05). These associations persisted even when controlled for variables that had significant associations with a lower Apgar score (OR = 1.3, 95% CI: 1.0-1.6, p < 0.05). Male fetuses were also more likely to stay in the NICU (1.0% vs 0.9%, OR = 1.1, 95% CI: 1.0-1.3, p < 0.05). After adjustment for confounders, the results still showed a significant association between male fetuses and a need for the NICU (OR = 1.3, 95% CI: 1.1-1.5, p < 0.001). Male fetuses were associated with a higher risk of neonatal death (0.40% vs 0.25%, OR = 1.6, 95% CI: 1.3-2.0, p < 0.01). After controlling for variables that significantly affected neonatal death, there were still significant differences between the two groups regarding this phenomenon (OR = 1.4, 95% CI: 1.1-1.8, p < 0.01) (Tables 4 and 5).

**Table 5:** **The relationship between fetal gender and neonatal outcomes**

**Deliveries**	**No of individual modes/total No**	**Crude OR (95% CI)**	**Adjusted OR (95% CI)**
Apgar < 7 at 5 min)^a^ (5346 missing cases for this outcomes)			
Female	193/47728	1	1
Male	294/56648	1.3 (1.1-1.5)^*^	1.3 (1.0-1.6)^*^
Admission to neonatal ICU^b^ (35 missing cases for this outcomes)			
Female	455/49675	1	1
Male	624/60012	1.1 (1.0-1.3)^*^	1.3 (1.1-1.5)^#^
Neonatal death^c^ (35 missing cases for this outcomes)			
Female	124/49675	1	0
Male	241/60012	1.6 (1.3-2.0)^#^	1.4 (1.1-1.8)^*^

*p < 0.05, ^*#*^ *p < 0.001.*

^a^Adjusted for grade of hospitals, resident, maternal education, primiparous, bad obstetrics history, modes of delivery, birth weight, HDCP, PROM, placenta previa, placenta abruption, other medical disorders, NRFHT, multiple fetus, malpresentation.

^b^Adjusted for maternal age, grade of hospitals, resident, maternal education, primiparous, bad obstetrics history, modes of delivery, birth weight, HDCP, PROM, placenta previa, placenta abruption, NRFHT, , multiple fetus,

^c^Adjusted for grade of hospitals, resident, maternal education, primiparous, bad obstetrics history, modes of delivery, birth weight, HDCP, PROM, placenta abruption, other medical disorders, NRFHT, multiple fetus, malpresentation.

5346 missing cases for Apgar scores at 5 minutes, 35 missing cases for neonatal ICU, 35 missing cases for neonatal death.

## Discussion

This retrospective hospital-based study had two main findings. First, our findings confirmed adverse pregnancy and labour outcomes for male fetuses. The second observation was an obvious neonatal sex bias in our survey.

It has been thoroughly documented that having a male fetus is associated with a higher rate of preterm labour. In addition, a higher prevalence of gestational hypertension in preterm males was previously reported and was suggested be an explanation for the phenomenon [10,11]. In contrast with previous studies, our study did not find higher incidences of HDCP and preeclampsia in male-bearing women or sex differences for GDM or PROM. Our results showed that male sex is an independent risk factor for preterm labour, even after adjustment for other significant factors that induce preterm birth. This finding suggests that the differences may be due to a biological mechanism.

Although several studies have suggested that pregnancies with male fetuses are associated with higher rates of fetal distress [12-14], an increased vulnerability to intrauterine hypoxia among male fetuses was not demonstrated in our study, and both the prevalence of NRFHT and the rate of caesarean section for NRFHT in male fetuses were lower than those in female fetuses. However, we found that male fetuses had an increased risk of a neonatal low Apgar score, need for NICU admission and neonatal death. These results were consistent with those of a previous study [5]. Previous study showed that the higher rates of GDM and HDCP in male fetuses were related to their adverse outcomes [3], therefore, we calculated the relative risk after adjusting for confounders. The results of the adjusted OR were almost the same as those for the crude OR. The coping abilities of male fetuses may be inferior to those of females, leading to increased risks of neonatal complications and death. Moreover, this difference was most pronounced for the high respiratory distress syndrome rate in male neonatal death [15], suggesting that a slower lung maturation among male fetuses contributes to the sex differences in neonatal mortality.

Several studies have suggested that pregnancies with male fetuses are associated with higher rates of labour dystocia [16-18]. The birth weight was considerably higher in male fetuses, this difference may account for the greater risk of caesarean or operative vaginal delivery due to dystocia [19,20]. However, one study reported that this phenomenon may be due to the larger head circumference of male infants [18]. Our study also showed that male fetuses were more likely than female fetuses to be macrosomic. Nonetheless, after adjustment for birth weight, the results still showed a significant association between male sex and caesarean section for dystocia. Additional factors beyond weight itself may cause the sex differences we have described.

Interestingly, FGR was more common among females in our study. Meanwhile, fetuses with a birth weight below 2500 g were more frequently female. The lower birth weights in these fetuses may account for the higher percentage of FGR. Several other studies have drawn similar conclusions, suggested that hormonal, physiological or gene factors may contribute to these phenomena [4,7]. The mechanism for this phenomenon remains largely unexplored.

It is noteworthy that our study demonstrated a male-to-female newborn ratio of approximately 1.2, which is much higher than the average international ratio (1.05). This unbalanced ratio of male-to-female births has persisted for nearly forty years [21]. Traditionally, women in Asian countries have a cultural preference for boys over girls, especially in rural China [22]. The cohorts in other countries have an excess of male perinatal deaths compared with female perinatal deaths, but in the Chinese cohort, females were more likely to die than males [23]. Female newborns can even increase the mother’s risk of postpartum depression in China [24]. Our results suggest an effect of different social and cultural environments on the sex ratio of newborns. Although sex identification of fetuses in China is banned by law, the unauthorised use of ultrasound in sex selection still occurs, and an increase in sex-selective abortions may be related to the obvious sex bias in China [25]. This phenomenon can induce severe social problems; thus, it should be paid attention by the Chinese government.

The high rate of maternal request for caesarean delivery in China was surprising. A study involving 56,968 caesarean deliveries showed that the prevalence rates of caesarean delivery during 1993–1995, 1996–2000 and 2001–2005 were 13.1%, 28.3% and 50.4% in southern China, respectively, and the prevalence rates of maternal request were 0.6%, 3.8%, and 12.9%, respectively [26]. Thereafter, the WHO conducted a study of 24 countries and reported that Chinese health facilities had the highest caesarean delivery rate of 46.2%, and had an 11.6% rate of caesarean deliveries without medical indications [27]. In our survey, the rate of maternal demand for caesarean delivery was 16.9% for all deliveries. Several clinical or non-clinical factors may explain the increasing rate of caesarean delivery without medical indications. Our results showed that the rate of maternal request for caesarean delivery among male-bearing women was slightly higher than that in female-bearing women, suggesting that the preference for male children remains in Chinese society.

The relationship between maternal age and fetal sex varied greatly in previous studies. Some reports have found a relatively lower proportion of male births with increasing maternal and paternal age [28,29]. However, among 549,048 births in Scotland, there was no association between maternal age and the offspring sex [30]. In contrast with the above study, we found that a great number of fetuses were female among women aged more than 35 years, which was also reported by Takahashi [31]. The present study also showed that primiparous mothers were more likely to deliver female fetuses. The male-to-female sex ratio may increase with increasing maternal gravidity and parity, and to the best of our knowledge, this phenomenon has not been observed previously. Further research is needed to clarify this observation.

## Conclusions

We concluded that a significant neonatal sex bias and male ‘disadvantage’ is present in our population. In maternal–fetal medicine, sex differences represent a novel parameter for prognostic information related to pregnancy and neonatal outcome. Our data may provide physicians with evidence to tailor their diagnoses of protraction and arrest disorders at the individual-patient level. We will perform more detailed studies in an attempt to more thoroughly understand the reasons for this phenomenon.

### Strengths and limitations

The major strength of this study is the study population size, which makes this study the largest-scale survey in China to date. We recognise that our study has some limitations. We did not select facilities randomly. Facilities were chosen from our cooperative centre, and the number of facilities was only approximately proportional to the regional population size; thus, the potential for selection bias exists. The results should not be regarded as representative rates and outcomes for the entire country. Furthermore, because our survey only included secondary and tertiary hospitals, it excluded people who live in certain rural areas and only have access to primary hospitals. Our results, especially the neonatal outcomes, may be better than those for primary hospitals. In addition, some useful information was missing, such as detailed labour progress descriptions, umbilical cord abnormalities, neonatal head and abdominal circumferences, and umbilical cord pH. We only had information about morbidities and mortality until time of discharge from the hospital, and therefore, some outcomes might be underestimated. Finally, the calculated odds ratio may overestimate the risk of adverse outcomes for the male sex. Although we adjusted for many potential confounding factors, there may have been other factors for which we had no information; thus, we could not adjust for these potential factors.

## Additional file

Additional file 1:
**The procedures of this study received ethics approval from the Human Ethics Committees of following hospitals.**
