# Supplementary material for: Cross sectional study in China: fetal gender has adverse perinatal outcomes in mainland China
Source: BMC Pregnancy Childbirth. 2014 Oct 26;14:372. doi: 10.1186/s12884-014-0372-4 (PMC4218998; doi:10.1186/s12884-014-0372-4)
Supplement: Additional file 1: — The procedures of this study received ethics approval from the Human Ethics Committees of following hospitals. [file 12884_2014_372_MOESM1_ESM.docx]

**Additional file 1**

The procedures of this study received ethics approval from the Human Ethics Committees of following hospitals

1. Capital Medical University, Beijing Obstetrics and Gynecology Hospital
2. Capital Medical University, Friendship Hospital
3. Beijing Daxing Maternal and Child Health Hospital
4. Tongzhou Maternal and Child Health Hospital
5. Inner Mongolia Maternal and Child Health Hospital
6. Erlianhaote People’s Hospital
7. Taiyuan Maternal and Child Health Hospital
8. Cangzhou Central Hospital
9. The First Affiliated Hospital of Inner Mongolia Medical University
10. Obstetrics and Gynecology Hospital of Fudan University
11. Shanghai Changning Maternal and Child Health Hospital
12. Shanghai Putuo Maternal and Child Health Hospital
13. Nanjing Drum Tower Hospital, The Affiliated Hospital of Nanjing University Medical School
14. Wuxi Maternal and Child Health Hospital
15. Shandong Provincial Hospital
16. Shandong Obstetrics and Gynecology Hospital
17. Dongming Country Maternal and Child Health Hospital
18. First Affiliated Hospital of Medical College of Xi’an Jiaotong University
19. Xi′an Aerospace General Hospital
20. Zichang Country People’s Hospital
21. Chenggu Country Maternal and Child Health Hospital
22. The First Affiliated Hospital of Xinjiang Medical University
23. Shengjing Hospital of China Medical University
24. Benxi Central Hospital
25. Kaiyuan Country People’s Hospital
26. Liaohe Youtian Maternal and Child Health Hospital
27. Xiuyan Country Maternal and Child Health Hospital
28. The Second Hospital Jilin University
29. Yushu Maternal and Child Health Hospital
30. Nongan Country People’s Hospital
31. Hubei Xinhua Hospital
32. The Eleventh Hospital of Wuhan
33. Wuhan commercial hospital
34. The Second Affiliated Hospital of West China Hospital，Sichuan University
35. Pengzhou Maternal and Child Health Hospital
36. Nanfang Hospital of Nanfang Medical University
37. The Third Affiliated Hospital of Nanfang Medical University
38. Shaoguan Maternal and Child Health Hospital
39. Fuoshan Maternal and the Child Health Hospital
